# Repurposing of PSMA-targeted diagnostic and therapeutic agents for the detection and treatment of giant cell tumors of bone

**DOI:** 10.3389/fonc.2024.1504514

**Published:** 2024-11-15

**Authors:** Brenna C. McAllister, Nooshin Mesbahi, Esther E. Dodson, Sakinah Abdulsalam, Clifford E. Berkman, Leslie A. Caromile

**Affiliations:** ^1^ Center for Vascular Biology, University of Connecticut Health Center, Farmington, CT, United States; ^2^ Department of Chemistry, Washington State University, Pullman, WA, United States; ^3^ Department of Neuroscience, University of Connecticut Health Center, Farmington, CT, United States

**Keywords:** prostate specific membrane antigen (PSMA), repurposable drugs, Pluvicto, Locametz, giant cell tumor of bone (GCTB)

## Abstract

Giant cell tumor of bone (GCTB) is a rare bone tumor often necessitating surgical intervention, radiation therapy, or treatment with bisphosphonates or denosumab. ^99m^Tc-MDP bone scintigraphy for GCTB has limited specificity, and the relatively high uptake of ^18^F-FDG in GCTB makes it challenging to differentiate it from other benign bone tumors. More specific detection and treatment modalities for GCTB are needed to enhance patient monitoring and outcomes. Prostate Specific Membrane Antigen (PSMA) is present in the neovasculature of various tumors, yet unexplored in GCTB. PSMA-targeted imaging and radiotherapeutic agents Locametz and Pluvicto are a powerful theranostic pair for detecting and treating PSMA-positive metastatic tumors, including those in bone, and thus have considerable potential to be repurposed for GCTB. This study aimed to determine if the vasculature of GCTB was PSMA-positive and whether targeting it with PSMA-specific agents was feasible. Using bone core samples from 28 GCTB patients and 9 negative controls, we present the first robust detection of PSMA on the tumor vasculature of GCTB. To demonstrate the potential repurposed use of PSMA-targeted agents in detecting and treating GCTB, we used a PSMA-specific fluorescent probe (FAM-C6-1298) as a model for these radiopharmaceutical agents. Incubation of fresh GCTB tissue samples with FAM-C6-1298 showed increased fluorescence intensity compared to controls, indicating successful targeting of PSMA in GCTB tissue. In conclusion, our data established that PSMA is not only present in the tumor vasculature of GCTB patient tissue but can be effectively targeted with repurposed PSMA-specific radiopharmaceuticals for diagnosis and therapy.

## Introduction

Giant cell tumor of bone (GCTB) is primary osteolytic neoplasm that accounts for approximately 5-6% of all primary bone tumors and about 20% of benign bone tumors (1). GCTB progression is driven, in part, by the overactivity of the receptor activator of nuclear factor-kappa β ligand (RANKL) (2) and typically affects the (3) decade of life (median age 20-40 years) (1, 4). The World Health Organization’s classification of soft tissue and bone tumors categorized GCTB as an intermediate malignant tumor with locally aggressive behavior and a high recurrence rate (5). GCTB has been observed to metastasize to the lungs in up to 6% of cases and can also undergo a malignant transformation in 2.4% of cases (6). The clinical presentation of GCTB includes local swelling, pain, and limitations in joint movement (7, 8). While ^99m^Tc-methyl diphosphonate (^99m^Tc-MDP) bone scintigraphy is routinely used for evaluating GCTB skeletal involvement, its utility is limited by reduced specificity (9). Additionally, ^18^F-fluorodeoxyglucose (FDG) uptake in GCTB, as measured by positron emission tomography/computed tomography (PET/CT), is comparatively higher than in other benign bone tumors due to the increased metabolic activity of osteoclasts (10, 11) making it difficult to differentiate between benign and malignant bone tumors. Unfortunately, a bone biopsy for histological examination is necessary for a final diagnosis of GCTB.

Due to the absence of randomized clinical trials (<50), treatment methods for GCTB have not significantly changed in the past three decades (12). The preferential treatment is curettage and high-speed drilling with local adjuvants and filling with polymethylmethacrylate (PMMA), bone allografts, and hydroxyapatite, often resulting in recurrence rates of 45% (1, 13–17). Where joint salvage is impossible, resection and reconstruction are favored. While joint replacements result in lower GCTB recurrence, they have higher complication rates and less favorable functional outcomes (18, 19). GCTBs that are inoperable, such as in the pelvis or spine, or cause severe dysfunction even after resection are treated with radiation therapy or antiresorptive drugs such as bisphosphonates and/or the human anti-RANKL antibody (denosumab). Bisphosphonates, such as zoledronic acid, function by inhibiting farnesyl pyrophosphate synthase, which is vital in promoting the attachment of the osteoclast to the bone. As a result, the osteoclast detaches from the bone surface, inhibiting bone resorption (20). Furthermore, Bisphosphonates inhibit osteoclast-like giant cell formation from immature precursors and induce apoptosis in mature osteoclasts. Though some literature supports the efficacy of bisphosphonates, the side effects are not trivial. In 15% - 30% of cases, patients experience nausea, fatigue, bone pain, hypotension, atrial fibrillation, anemia, and alopecia, to name a few. More severe cases include osteonecrosis of the jaw (3).

Denosumab is a human monoclonal antibody that binds the cytokine RANKL, an essential factor initiating bone turnover. RANKL inhibits monocyte activation and osteoclastogenesis, thus reducing bone resorption (21, 22). The response rate to denosumab, defined as more than a 90% depletion of multinucleated giant cells on histopathologic examination, is approximately 72% (23, 24). However, caution is employed since upwards of 40% of recurrent GCTB that transform into malignant sarcomas are found in patients who received denosumab administration before curettage for their initial benign lesion (25, 26). Additionally, denosumab cessation carries a risk of relapse, thus requiring long‐term treatment resulting in serious adverse effects (25, 27–29). Therefore, despite efforts, there is a lack of specific detection and treatment methods to improve patient monitoring and reduce bone-related events for GCTB patients. However, if a clinically relevant biomarker for other indications could be identified in GCTB cells, it could support the feasibility of repurposing relevant drugs targeted to such a biomarker.

Prostate-Specific Membrane Antigen (PSMA) is the hallmark enzyme-biomarker for prostate cancer as it is expressed in the epithelium of nearly all prostate cancers, and increased expression correlates with progression to castration resistance and metastatic disease (30–32). PSMA is a type II transmembrane protein with glutamate-carboxypeptidase activity and known substrates. Upon ligand binding, the cytoplasmic domain of PSMA contains an *N-*terminal motif that signals the internalization of PSMA via clathrin-coated pits (33, 34), resulting in the transportation of bound ligands into the cell. Clinical technologies utilize this signaling pathway to enhance tumor detection and management of prostate cancer through the delivery of radiopharmaceuticals into primary and metastatic prostate tumors, with PSMA-targeted PET ([^68^Ga] Ga-PSMA-11 (Locametz)) and ([^177^Lu] Lu-PSMA-617 (Pluvicto)) leading the way (35–45). Tumor vascularity significantly impacts tumor growth and drug responsiveness concerning tumor oxygenation and permeability of imaging agents and chemotherapeutics (46–49). In addition to its unique expression in prostate cancer, PSMA is known to be expressed on the endothelial cells of neovasculature in both prostatic and non-prostatic tumors (e.g., renal cell carcinoma, and breast, lung, gastric, colorectal, pancreatic, and bladder cancers) (44, 50–52). However, to date, there have been no reports on the expression of PSMA in the vasculature of GCTB nor any on PSMA-based detection or treatments regarding this disease. If the vasculature of GCTB was similarly characterized by PSMA expression, there would be sufficient rationale for pursuing the repurposing of clinical PSMA-targeted diagnostic and therapeutic agents such as Locametz and Pluvicto.

Drug repurposing involves identifying new therapeutic uses for existing drugs initially developed for other indications. A drug’s specific pharmacological action frequently gives rise to a spectrum of side effects, which may exhibit secondary therapeutic uses. Drug repurposing has several advantages over developing new drugs, including a lower risk of failure due to established safety profiles, reduced development time, and lower investment requirements (53–57). Repurposable drugs include generic (off-patent) medications currently available on the market, on-patent medications such as Locametz and Plavicto, including those still undergoing clinical trials, and failed drugs initially intended for a different purpose. New potential medication applications are often uncovered through pre-clinical *in vitro and in vivo* experiments, mathematical modeling, AI-driven network simulations, and clinical trials (58, 59). This strategic approach has primarily targeted chronic conditions such as diabetes, cancer, and rare diseases.

Our aim in this study was to determine if the vasculature of GCTB was positive for PSMA and, if so, whether it would be feasible to target it with PSMA-specific small-molecule fluorescent probe (45). In our analysis of samples obtained from patients clinically diagnosed with GCTB, we detected a significant presence of PSMA on the endothelial cells of tumor vasculature compared to the control. Furthermore, our results demonstrated the effective internalization and trafficking of a model PSMA-targeted agent into a PSMA (+) human cell line and the targeting of PSMA in GCTB patient tissue. This finding is of substantial clinical importance, especially given the recent availability of the PSMA-targeted radiopharmaceuticals Pluvicto and Locametz and their broader applicability for indications other than prostate cancer. This proof-of-concept paper supports the feasibility of initiating preclinical studies and randomized clinical trials focusing on the repurposing of commercially available PSMA-targeted diagnostic and therapeutic agents for the detection and treatment of GCTB.

## Materials and methods

### Cells

The immortalized human prostate cancer cell line C4-2B (ATCC, Manassas, VA) used in this study was maintained in RPMI 1640 medium (Thermo Fisher, Waltham, MA), supplemented with 10% fetal bovine serum, 100 µg/ml antibiotic-antimycotic (Thermo Fisher), and insulin-transferrin-selenium (Thermo Fisher) in a humid atmosphere containing 5% CO_2_ at 37°C.

### Immunohistochemistry

Clinically diagnosed, deidentified, and coded GCTB patient bone core formalin-fixed paraffin-embedded (FFPE) slides were obtained commercially from TissueArray.com (catalog numbers BO801, BO601, and T261b). An in-house pathologist from TissueArray.com and/or the clinical source verified the clinical diagnosis of GCTB using H&E staining and IHC with Anti-S-100 and H3.3. Slides were deparaffinized and rehydrated. Antigen retrieval was conducted at 95°C using 10 mM sodium citrate buffer (pH 6.0, EMD Millipore Corp. Burlington, MA) in a steamer. Endogenous peroxidase activity was quenched by incubating slides for 15 mn in a peroxidase suppressor (Thermo Fisher). Slides were blocked in 10% normal goat serum in PBS for 60 mn at room temperature in a humidified chamber and then incubated with PSMA rabbit monoclonal antibody (Cell Signaling Technology, Danvers, MA) or a CD31 mouse monoclonal antibody (Cell Signaling Technology) 1% normal goat serum, and 1X PBS overnight at 4°C in a humidified chamber. Slides were washed in PBS, and VECTASTAIN Elite ABC Universal PLUS Peroxidase Kit (anti-mouse/rabbit IgG) (Vector Laboratories, Newark, CA) was used according to the manufacturer’s instructions. Slides were developed using 3,3’-diaminobenzidine. Slides were counterstained in Hematoxylin Gill’s Formula (Vector Laboratories), differentiated in a 1% acetic acid rinse, followed by a bluing solution, and then rehydrated and mounted under Cytoseal 60 (Epredia, Kalamazoo, MI). Images were acquired using a Zeiss LSM510 META based on an Axiovert 200 microscope and processed using the Zeiss Zen software v3.6. Representative H&E images for each patient can be found on the TissueArray.com website.

### Immunofluorescence staining

Commercial clinically diagnosed, deidentified, and coded FFPE bone core slides (TissueArray.com, catalog #BO601, #BO801, and #T261b) were deparaffinized and rehydrated. Antigen retrieval was conducted at 95°C using 10 mM sodium citrate buffer, pH 6.0 (EMD Millipore Corp) in a steamer. Slides were blocked and permeabilized in 0.01% Triton X-100 and 10% normal goat serum (Sigma Aldrich, St. Louis, MO) for 1 h and then incubated with a PSMA rabbit monoclonal antibody (Cell Signaling Technology) and a CD31 mouse monoclonal antibody (Cell Signaling Technology), in 1% normal goat serum overnight in humidity chamber 4°C. Slides were washed in PBS and incubated with Alexa Fluor 488 goat anti-mouse (Thermo Fisher Scientific) and Alexa Fluor 546 goat anti-rabbit (Thermo Fisher Scientific) for 1 h. Slides were washed in PBS, and autofluorescence was quenched with Vector TrueVIEW (Vector Laboratories). Slides were washed in PBS and mounted in VECTASHIELD Hardset Antifade Mounting Medium with DAPI (Vector Laboratories.). Images were acquired using a Zeiss LSM510 META based on an Axiovert 200 microscope and processed using the Zeiss Zen software v3.6.

### FAM-C6-1298 synthesis

The synthetic methods for preparing FAM-C6-1298 are detailed in the **Supplementary Information**. DBCO-C6-1298 was available from a prior study (60) and 5-FAM-azide was purchased from Lumiprobe Corporation. All other reagents and general solvents were of commercial quality (Fisher Scientific, Sommerville, NJ) or (Sigma-Aldrich, St. Louis, MO) and were used without further purification. Anhydrous solvents used in reactions were obtained from commercial sources or freshly distilled over calcium hydride. ^1^H, ^13^C, and ^31^P NMR spectra were recorded on a Varian 400, Brucker Avance Neo 500, or Varian 600 MHz spectrometer. ^1^H NMR chemical shifts are relative to CDCl_3_ (δ = 7.26 ppm), CD_3_OD (δ = 3.31 ppm) or D_2_O (δ = 4.79 ppm). ^13^C NMR chemical shifts were relative to CDCl_3_ (δ = 77.23 ppm) or CD_3_OD (δ = 49.15 ppm). ^31^P chemical shifts were relative to triphenylphosphine oxide (TPPO, δ = 27.00 ppm). High-resolution mass spectrometry (HRMS) spectra were obtained on an Applied Biosystems 4800 MALDI-TOF/TOF mass spectrometer (Applied Biosystems, Foster City, CA).

### Fluorescent cell imaging

Cells were plated onto coverslips at a density of 1 × 10^5^ cells/well in growth medium and allowed to attach for 48 h. Cells were then incubated for 30 min with either 100μM DBCO-C6-1298 or a control growth medium, followed by 30 min with 10μM FAM-C6-1298 at 37°C. For imaging, coverslips were set on ice, rinsed three times in ice-cold phosphate-buffered saline (PBS), fixed in ice-cold 10% neutral buffered formalin solution for 15 min, rinsed again with PBS, and mounted in VECTASHIELD Hardset Antifade Mounting Medium with DAPI (Vector Laboratories).

### Fluorescent co-localization cell imaging

Cells were plated onto coverslips at a concentration of 1 × 10^5^ cells/well in 1 mL of growth medium and allowed to attach overnight. Cells were starved for 2 h in fetal bovine serum (FBS)-free RPMI and then incubated for 60 min with 1μM FAM-C6-1298. Coverslips were set on ice, rinsed twice in ice-cold phosphate-buffered saline (PBS), and fixed in ice-cold 10% neutral buffered formalin solution for 15 min. Coverslips were blocked and permeabilized in 0.01% Triton X-100 and10% normal goat serum (Sigma) for 1 h and then incubated with PSMA antibody (D718E, Cell Signaling Technology) in 1% normal goat serum overnight in a humidity chamber 4°C. Coverslips were washed in PBS and incubated with Alexa Fluor 546 goat anti-rabbit (Thermo Fisher Scientific) for 1 h. Slides were washed in PBS and mounted in VECTASHIELD Hardset Antifade Mounting Medium with DAPI (Vector Laboratories).

### *Ex vivo* whole-tissue fluorescence measurements

Frozen tissue blocks from clinically diagnosed GCTB deidentified and coded patients were purchased commercially from OriGene (OriGene, catalog number CB499383, CB649405), divided into two sections, and washed in DPBS at 37°C for 10 mn and then HEPES at 37°C for 10 mn. Tissue blocks were then incubated with either 10μM FAM-C6-1298 in HEPES for 60 min or with 100μM DBCO-C6-1298 in HEPES for 60 min, followed by a 60 min incubation with 10μM FAM-C6-1298 in HEPES at 37°C. The tissue was washed three times in DPBS, placed in 60mm plates, and analyzed for fluorescence using the IVIS Spectrum 2 Imaging System (Revvity). The fluorophore of FAM-C6-1298 was excited at 495 nm and detected at 517 nm. Data was collected as radiant efficiency (photons/sec/cm^2^/steradian/μW/cm^2^) using Living Image software v4.8.2. To mitigate the effects of arbitrary autofluorescence, the software computes the background-corrected intensity signal using the following formula:


Background corrected intensity = ROI intensity − Average background ROI intensity

### Reagents

All reagents used in this project can be found in the **Supplementary Materials**.

### Ethical statement

All patient samples used in this study were purchased commercially from TissureArray.com (Derwood, MD) and OriGene (Rockville, MD). The following links provide information on the HIPPA-compliant tissue collection procedures and ethical standards followed by these companies:

https://www.tissuearray.com/FAQs#q10

https://www.origene.com/products/tissues/tissue-qc

### Statistical analysis

Differences between means were analyzed using either the two-tailed Student’s t-test or analysis of variance (ANOVA), where appropriate, and significance was set at p ¾ 0.05. NIH/FIJI was used to analyze IF co-localization staining. The Zeiss Zen software v3.6 co-localization function determined the Pearson correlation coefficient (ρ).

## Results

**Figure 1:**
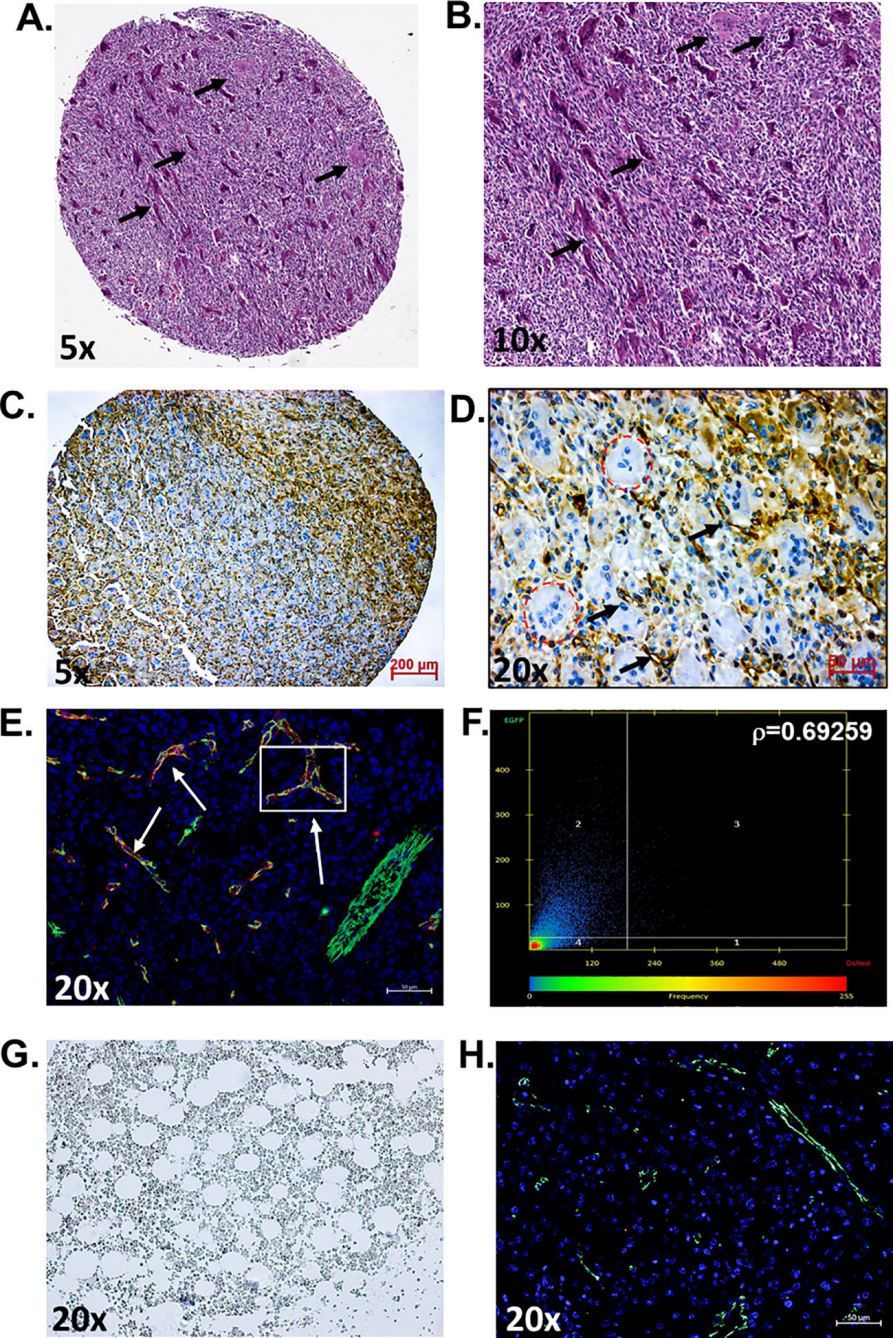
PSMA is detected on the vasculature of GCTB. **(A, B)** Representative hematoxylin and eosin staining of an FFPE bone biopsy core from a 20-year-old male with clinically diagnosed GCTB. Arrows point to multinucleated giant cells that are a hallmark of GCTB (black arrows). **(C, D)** IHC of an FFPE bone biopsy from a 20-year-old male with GCTB is positive for PSMA, as visualized by the brown precipitate and black arrows. Multinucleated giant cells counterstained with hematoxylin can be identified within the section (circled in red). **(E)** IF staining of PSMA (red), CD31 (green), and the nucleus (blue, DAPI). White arrows indicate examples of Co-localization. **(F)** Pearson correlation coefficient (r) as measured by Zeiss Zen software. The intensity of a given pixel in the CD31 image is used as the y-coordinate of the scatter plot, and the intensity of the corresponding pixel in the PSMA is the x-coordinate. **(G)** Cancer adjacent normal bone and bone marrow tissue (NAT) of rib containing a layer of adipocytes adjacent to bony trabeculae and red blood cells were used as negative controls and is void of PSMA staining. **(H)** IF staining of NAT negative control for PSMA (red) and CD31 (green), nuccleus (blue, DAPI) (20x, scale bar = 50μM). N=28 GCTB patient bone core samples, N=9 NAT controls. The immunofluorescence and immunohistochemistry images, as well as the co-localization information for all 28 patients and negative controls, can be found in **Supplementary Figures 1**–**3**. All tissue in this figure was purchased from MicroArray.Com, and further information about the tissue can be found in Materials and Methods. Hematoxylin and eosin images are from TissueArray.Com.

In this study, we aimed to determine if the vasculature of GCTB was positive for PSMA and, if so, whether it would be feasible to target it with a PSMA-specific small-molecule fluorescent probe (45). Deidentified and coded GCTB patient samples were obtained commercially from TissueArray.com. The in-house pathologist from the company and/or the clinical source verified the clinical diagnosis of GCTB using hematoxylin and eosin staining (**Figures 1A, B**) and IHC with Anti-S-100 and/or H3.3 (not provided). Multinucleated giant cells can be identified within the tumor tissue by hematoxylin and eosin counterstaining, which is a key characteristic of GCTB (61). For our analysis, GCTB patients were chosen to represent a wide range of ages to assess the broad applicability of PSMA agents for GCTB and prevent sampling bias. Therefore, in our immunohistochemical examination of FFPE bone core samples from 28 patients (12 female, 16 males, ages 17y-75y) clinically diagnosed with GCTB and 9 negative control FFPE patient bone core samples from cancer adjacent normal bone (NAT) of rib (five female, four male, ages 50y-68y) (**Table 1**), we present the first robust detection of PSMA on the tumor vasculature of GCTB compared to NAT control (**Figures 1C, D**). Additionally, to validate that PSMA was restricted to the endothelial cells of the GCTB vasculature, we co-incubated the FFPE bone core samples with the vascular endothelial cell marker CD31 and PSMA. Using a previously published Pearson correlation coefficient (ρ) scale where a correlation of <0.20 is very weak, a correlation between 0.20-0.39 is weak, correlations between 0.40-0.59 are moderate, correlations between 0.60-0.79 are strong, and correlations >0.80 as very strong (62), we found that 71.4% of GCTB samples exhibited a strong to moderate positive fluorescent co-localization (ρ = 0.5 to 0.7) of CD31 and PSMA compared to control NAT samples (**Figures 1E, G, H**), thus confirming that PSMA was restricted to the endothelial cells of the GCTB vasculature. The linear relationship between PSMA and CD31 fluorescent co-localization was verified through a scatterplot (**Figure 1F**). Due to section variability and random vasculature location within the tumor, the Pearson correlation analysis was measured within a specified boxed area on the image. However, the Pearson correlation coefficient cut-offs are set arbitrarily to refer to linear associations, which do not always exist (62). Therefore, to validate our data further and obtain a more accurate measurement of co-localization throughout the entire section, we used FIJI/Image J to calculate the area of PSMA co-2localization as a percentage of the area of CD31 staining using 10% as our cutoff for PSMA positive staining (**Table 1**; **Supplementary Table 1**). We found tissue sections with positive PSMA staining in the vasculature, and their corresponding ρ-values aligned with percentages greater than 10%. Two patient samples displayed disagreement between the Pearson coefficient and percent area, highlighting the influence that section variability and vasculature location within the GCTB have on these measurements. Taken together, analysis (visual assessment, Pearson correlation coefficient, and percentage) confirms that PSMA-positive staining is restricted to the vasculature of GCTB. The immunofluorescence and immunohistochemistry images, as well as the co-localization information for all 28 patients and negative controls, can be found in **Supplementary Figures 1**–**3**. When patient samples were stratified by biological sex, 87.5% of males were positive for PSMA tumor vasculature staining, compared to 50.0% of female samples (**Table 1**). While intriguing, additional validation is required to formulate any definitive conclusions.

**Table 1:** Patient biopsy information and GCTB PSMA vasculature staining status.

	Age	Sex	Organ	Site	Pathology Diagnosis	Grade	PSMA Staining	ρ	Percent Area
Female									
1	17	F	Bone	Left femur	GCTB	I	+	0.68695	52.33
2	24	F	Bone	Right humerus	GCTB	I	+	0.64367	44.51
3	32	F	Bone	Inferior left femur	GCTB	I	-	0.17155	2.14
4	33	F	Bone	Right femur	GCTB	I	-	0.27750	2.58
5	36	F	Bone	Right Femur	GCTB	II	+	0.62276	10.11
6	37	F	Bone	Ilium	GCTB	I	+	0.64784	17.91
7	38	F	Bone	Right humerus	GCTB	I	+	0.63026	19.83
8	38	F	Bone	Left wrist	GCTB	I	+	0.73253	50.55
9	40	F	Bone	Distal radius	GCTB	I	-	0.13626	3.95
10	42	F	Bone	Humerus	GCTB	I	-	0.20002	2.35
11	45	F	Bone	Left tibia	GCTB	I	-	0.28878	3.22
12	48	F	Bone	Left femur	GCTB	I	-	0.10619	4.97
Male									
1	20	M	Bone	Right femur inferior	GCTB	I	+	0.69259	15.41
2	20	M	Bone	Left fibula	GCTB	I	-	0.17428	1.37
3	22	M	Bone	Left femur	GCTB	II	+	0.55392	9.23**
4	28	M	Bone	Left femur/clavicle	GCTB	II	+	0.68700	31.19
5	30	M	Bone	Inferior right tibia	GCTB	I	+	0.68068	12.65
6	32	M	Bone	Inferior right femur	GCTB	I	+	0.64174	18.16
7	32	M	Bone	Left tibia superior	GCTB	I	-	0.49351	3.82**
8	32	M	Bone	Bone	GCTB	I	+	0.70681	16.99
9	32	M	Bone	Superior left tibia	GCTB	I	+	0.64874	16.35
10	33	M	Bone	Right humerus	GCTB	I	+	0.67971	58.14
11	33	M	Bone	Right humerus	GCTB (necrosis)	*	+	0.61930	58.39
12	34	M	Bone	Right femur and sacrum	GCTB	II	+	0.67971	56.99
13	34	M	Bone	Right leg	GCTB	II	+	0.62412	12.66
14	47	M	Bone	Left tibia superior	GCTB	I	+	0.65317	14.85
15	50	M	Bone	Right femur inferior	GCTB	I	+	0.65802	25.24
16	75	M	Bone	Left distal radius	GCTB	I	+	0.64481	38.49
Negative Controls									
1	50	F	Bone	Rib	NAT		-	NA	NA
2	50	F	Bone	Rib	NAT		-	NA	NA
3	56	F	Bone	Rib	NAT		-	NA	NA
4	68	F	Bone	Rib	NAT		-	NA	NA
5	68	F	Bone	Rib	NAT		-	NA	NA
6	56	M	Bone	Rib	NAT		-	NA	NA
7	60	M	Bone	Rib	NAT		-	NA	NA
8	63	M	Bone	Rib	NAT		-	NA	NA
9	66	M	Bone	Rib	NAT		-	NA	NA

Grade as measured by the Campanacci grading system: Grade I: A latent lesion with a well-defined margin and an intact cortex; Grade II: An active lesion with a relatively well-defined margin but no radiopaque rim; Grade III: An aggressive lesion with indistinct borders and cortical destruction (13). *Indicates no grading scale. ρ = Pearson correlation coefficient: Correlations <0.20 as very weak, correlations between 0.20-0.39 as weak, correlations 0.40-0.59 as moderate, correlations 0.60-0.79 as strong, and correlations >0.80 as very strong (62). The percent area co-localization was determined using the color threshold function in ImageJ/Fiji. The area of PSMA co-localization was reported as a percentage of the area of CD31 staining, and the average percentage was reported from serial sections. If >10% PSMA positive (+) staining. **Indicates disagreement between the percent area and r correlation coefficient due to section variability. Cancer adjacent normal bone and bone marrow of rib (NAT) were used as negative controls.NA, Not Applicable.

To illustrate the potential use of PSMA-targeted therapeutics in detecting and treating GCTB, we utilized a PSMA-specific small-molecule fluorescent probe, FAM-C6-1298, as a model. The structure of FAM-C6-1298 is derived from CTT1298 (developed by our lab), which binds *irreversibly* to enzymatically active PSMA and rapidly internalizes into PSMA (+) cells (36, 63). When derivatized, CTT1298 and its congeners possess nanomolar affinity and can deliver a diverse array of payloads (MMAE, SN38, doxorubicin, therapeutic radionuclides, therapeutic enzymes) into the cell (42, 64–74). The specificity and affinity of FAM-C6-1298 are analogous to that of the radiopharmaceuticals Locametz and Pluvictor. Here, we used FAM-C6-1298 as a model PSMA-targeting agent due to its ease in microscopic visualization (**Supplementary Figure 4**). After binding to extracellular PSMA, CTT1298-based conjugates rapidly traffic to endosomes/lysosomes through the internalization of the PSMA-conjugate complex (42, 45, 69–71, 75). We have previously confirmed that CTT1298 derivatives are internalized 99% in PSMA (+) cells within 4 h (38, 39).

**Figure 2:**
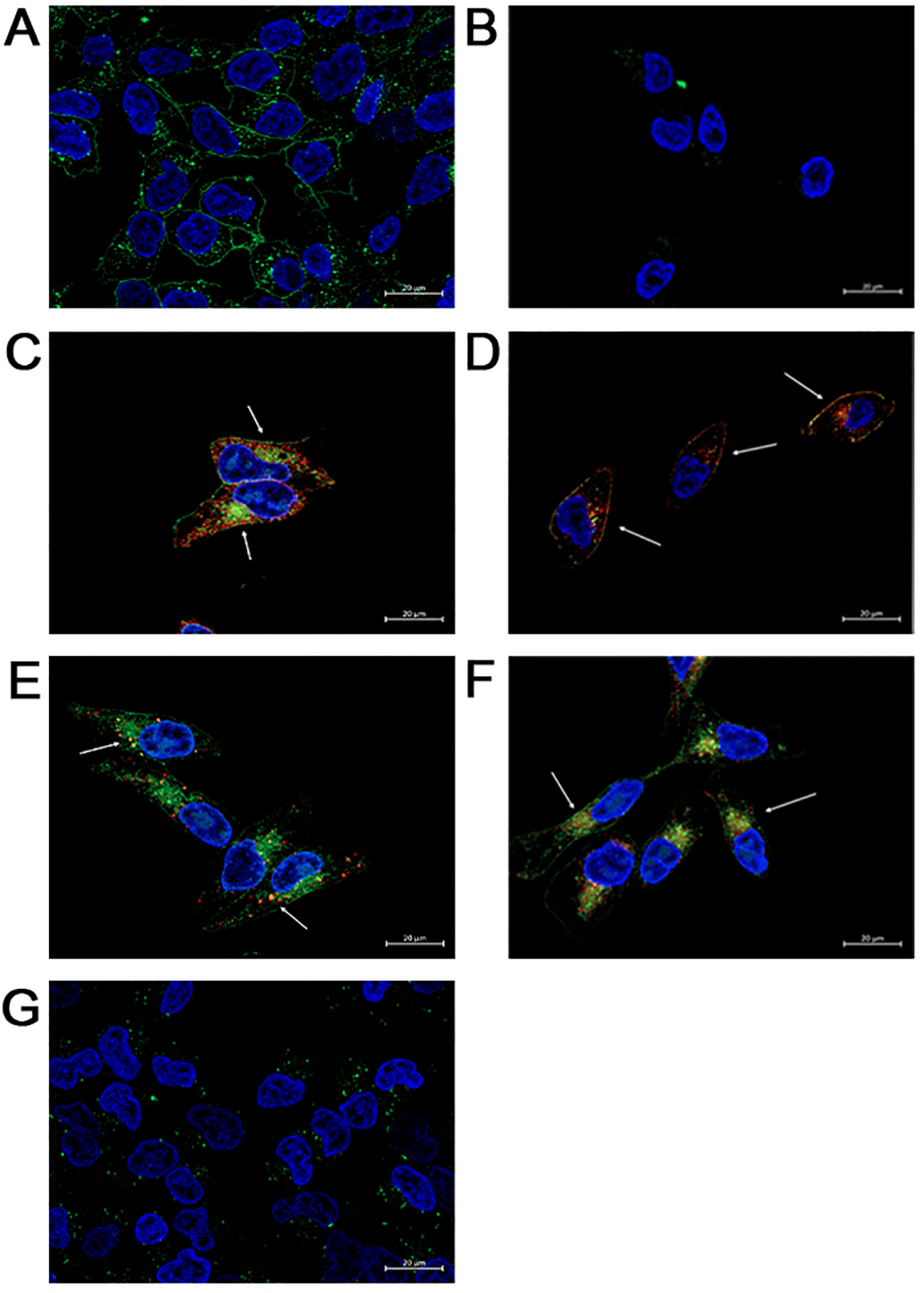
FAM-C6-1298 can bind to the cell surface PSMA of C42B cells and be internalized. **(A, B)** C42B-CRISPR-PSMA^scramble^ and C42B-CRISPR-PSMA^knockout^ cells incubated with 10mM FAM-C6-1298 for 30 mn. **(C)** Co-localization of FAM-C6-1298 (green) and PSMA (red) in C42B cells. **(D)** IF staining of positive control PSMA (red) and 5FAM-X-FPO-42 (green). White arrows point to areas of co-localization. **(E)** Co-localization of FAM-C6-1298 (green) and EEA1 (red) in C42B cells **(F)** Co-localization of FAM-C6-1298 (green) and LAMP-1 (red) in C42B cells. **(G)** C42B cells were incubated with 100μM DBCO-C6-1298 PSMA blocking peptide for 30 mn and then 10μM FAM-C6-1298 for 30 mn. 63x oil, scale bar = 20μM. The cell nucleus is stained with DAPI in all images. All experiments were repeated for at least three independent experiments.

To specifically establish that FAM-C6-1298 was suitable for addressing our experimental question, we confirmed that FAM-C6-1298 could bind to the cell surface PSMA of a PSMA-positive cell line and that the PSMA-bound FAM-C6-1298 could be internalized into the cell through the endosome-lysosome pathway. Using our previously described PSMA CRISPR knockout human prostate cancer C42B cell line (45), we treated C42B-CRISPR-PSMA^scramble^ and C42B-CRISPR-PSMA^knockout^ with 10μM FAM-C6-1298 for 30 min. Fluorescence microscopy indicated that FAM-C6-1298 bound to cell surface PSMA of the C42B-CRISPR-PSMA^Scramble^ cells and not C42B-CRISPR-PSMA^knockout^ (**Figures 2A, B**). Positive co-localization of FAM-C6-1298 and a PSMA antibody (52.16%) (**Figure 2C**) was comparable (64.55%) to our previously published PSMA-targeted probe 5FAM-X-FPO-42, which served as the positive control (**Figure 2D**) (45), further supporting the specificity of current PSMA-targeted imaging probe FAM-C6-1298. Additionally, fluorescence microscopy of C42B-Crispr-PSMA^Scramble^ cells indicated that the PSMA-FAM-C6-1298 complex co-localized with 10.49% of the early endosomal marker, EEA1, and the 55.04% with the lysosomal marker LAMP-1, providing evidence that the PSMA-FAM-C6-1298 complex was internalized into the cell (**Figures 2E, F**). The results of the percent co-localization measurements can be found in **Supplementary Table 2**.

To confirm that FAM-C6-1298 bound solely to cell surface PSMA, we first treated both C42B-CRISPR-PSMA^scramble^ and C42B-CRISPR-PSMA^knockout^ with 100μM of a previously published non-fluorescent PSMA-blocking ligand (DBCO-C6-1298) (76). This peptide binds exclusively to and blocks the enzymatic domain of extracellular, membrane-bound PSMA. After a 30-minute incubation with the blocking peptide, we further incubated the cells with 10μM FAM-C6-1298 for an additional 30 min. As predicted, no binding of FAM-C6-1298 was detected by fluorescent microscopy (**Figure 2G**). Therefore, the PSMA-targeted small-molecule fluorescent analog of Pluvicto and Locametz, FAM-C6-1298, demonstrated specific binding to PSMA, and the PSMA-FAM-C6-1298 complex was internalized and trafficked through the endosomal-lysosomal pathway, indicating its suitability as a therapeutic model for addressing our experimental question.

**Figure 3:**
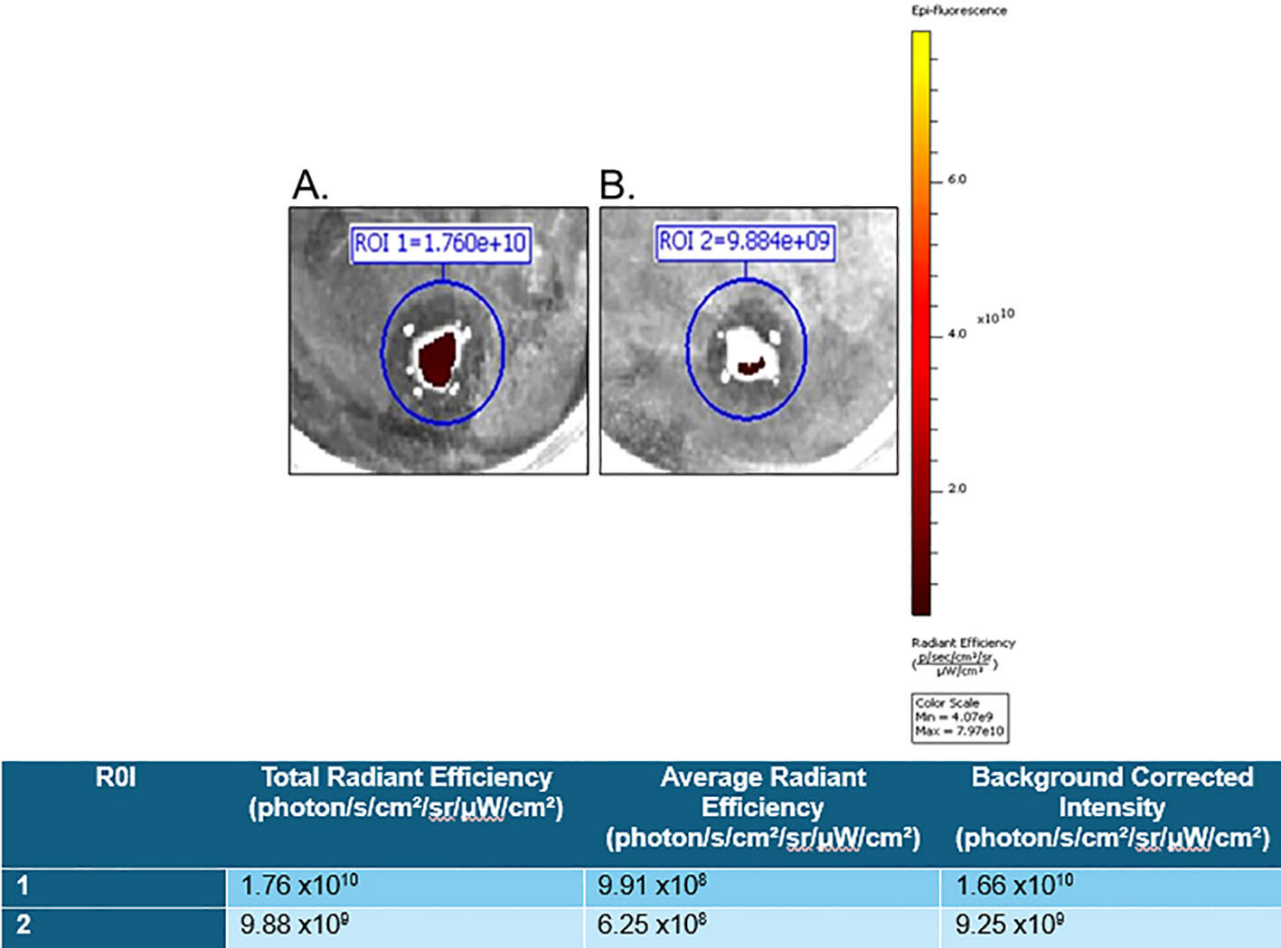
FAM-C6-1298 can successfully target PSMA in GCTB tissue. *Ex vivo* whole-tissue fluorescence measurements of fresh tissue from the tibia of a 23-year-old female with GCTB (OriGene, catalog number CB499383) incubated with **(A)** 10μM PSMA-targeted fluorescent probe, FAM-C6-1298. **(B)** 100μM PSMA blocking peptide DBCO-C6-1298, followed by incubation with 10μM FAM-C6-1298. Data was collected by IVIS as radiant efficiency (photons/sec/cm^2^/steradian/μW/cm^2^) using Living Image software v4.8.2 and presented as a background-corrected intensity signal in the chart. N=2. Information for sample 2 is in **Supplementary Figure 6**. All tissue in this figure was purchased from OriGene, and further information about the tissue can be found in Materials and Methods.

For proof of the *in vitro* applicability of a PSMA-targeted therapeutic in detecting and targeting GCTB, we commercially obtained fresh frozen tissue samples from patients clinically diagnosed with GCTB from OriGene (patient information in **Supplementary Figure 5**) and incubated them with either 10μM of our model PSMA-specific small-molecule fluorescent probe (FAM-C6-1298) alone or with 100μM of the PSMA-blocking ligand DBCO-C6-1298, followed by incubation with 10μM FAM-C6-1298. Tissue samples incubated with the combination of the PSMA-blocking peptide and FAM-C6-1298 displayed drastically decreased radiant efficiency (9.25x10^9^ photons/sec/cm^2^/str/μW/cm^2^) (**Figure 3B**), as measured by IVIS compared to those treated with the FAM-C6-1298 alone (1.66x10^10^ photons/sec/cm^2^/str/μW/cm^2^) (**Figure 3A**). Data for additional samples can be found in **Supplementary Figure 6**. Taken together, our data indicate successful targeting specificity and uptake of the model PSMA-targeted agent in GCTB tissue.

## Discussion

Drug discovery and development are critical processes in improving human health. The conventional drug development process typically involves several stages, including target identification, compound screening, preclinical studies, clinical trials, and regulatory approval. Unfortunately, this process is often slow, costly, and has high failure rates due to safety or efficacy issues. The average investment for developing a new drug is more than $2.5 billion, and it takes 10-15 years (53, 54) for a new product to be developed, with less than 10% of Phase I candidates receiving FDA approval (55, 56). An alternative strategy known as “drug repurposing” has gained traction to address these challenges and accelerate the discovery of new treatments. Because a drug already has an established safety profile, drug repurposing often skips Phase 1, advancing to Phases 2 and 3 with fewer pharmacokinetic uncertainties, thus significantly reducing the time and costs associated with the drug development process (56, 57). This ultimately results in improved patient outcomes.

Interestingly, in June 2010, denosumab - a treatment for GCTB - was initially approved by the FDA for non-cancer use in postmenopausal women with the risk of osteoporosis under the name Prolia (77), and repurposed in November 2010 as Xgeva for the prevention of skeleton-related events in patients with bone metastases from solid tumors, including prostate cancer (78, 79). In the summer of 2011, clinical trials investigated the safety and efficacy of denosumab in giant cell tumors, multiple myeloma with bone metastases, and hypercalcemia of malignancy (24, 26, 80). In June 2013, the FDA expanded the approved use of Xgeva to treat adults and some adolescents with GCTB. While denosumab’s efficacy in treating advanced and unresectable tumors is well-established, its role in managing surgically resectable disease is a topic of ongoing debate (22).

GCTB is prone to several factors that can impede successful treatment. Research has indicated that delayed diagnosis and treatment of GCTB are associated with increased tumor size, heightened recurrence rates, and elevated incidences of local complications. Moreover, patients experiencing delayed diagnosis or treatment are more likely to require aggressive interventions such as amputation or chemotherapy (81). In terms of location, sacral GCTB warrants special attention. Despite being one of the commonly affected bones, the treatment for sacral GCTB remains challenging, as sacrificing sacral nerve roots is associated with severe morbidity, such as the disturbance of gait and foot plantar flexion, as well as bowel and bladder dysfunction. Even after successful nerve-sparing surgery, the high recurrence rate (25-35% in most cases and up to 50% in some studies) often demands additional therapy (82, 83). Thus, there is an urgent clinical need to identify and develop novel therapeutic strategies for these patients.

PSMA-targeted radiopharmaceuticals, such as Pluvicto and Locametz, represent a potentially powerful theranostic combination for the detection and selective treatment of PSMA-positive GCTB vasculature. By leveraging the high expression of PSMA in the surrounding tumor vasculature, it is expected that these agents can precisely pinpoint and deliver therapeutic payloads to PSMA-expressing tumor vascular endothelial cells. Therefore, PSMA-targeted treatment in combination with traditional or surgical intervention (where possible) could be highly effective compared to a single therapeutic approach.

Through the use of a PSMA-targeted small-molecule fluorescent analog of Pluvicto and Locametz, we showed that PSMA-targeted agents offer a potential alternative to the detection and treatment of tumor vasculature in GCTB. One limitation of this study is the small sample size of 28 GCTB patients. Consequently, it is imperative to conduct further studies with sufficiently large sample sizes to ensure the replicability and generalizability of our findings. However, we still believe that this finding is timely and of substantial clinical importance, especially given the recent availability of Pluvicto and Locametz and their broader applicability for indications other than prostate cancer. While others are currently working on the identification of biomarkers as potential predictive indicators or druggable targets to improve management of GCTB (84), to date, there have been no reports on the expression of PSMA in the vasculature of GCTB or in any other primary bone cancer, such as osteosarcoma, Ewing sarcoma, chondrosarcoma, or chordoma. Indeed, Heitkötter et al. used immunohistochemistry to show that PSMA was present in Ewing sarcoma tumors. However, they did not convincingly establish that PSMA co-localized with endothelial cells of the tumor vasculature (85). While Parihar et al. demonstrated that Ga-PSMA-HBED-CC PET/CT described high radiotracer activity in the iliac bone of a single Ewing sarcoma patient (86) and Can et al. reported high radiotracer activity of ^68^Ga PSMA PET/CT in the primary tumor and metastatic lesions of a 75-year-old man with osteosarcoma of the sternum (87), neither established that PSMA was present on the endothelial cells of the tumor vasculature of these primary bone tumors.

In conclusion, there is potential for repurposing the current commercially available clinical PSMA-targeted agents for the detection and treatment of GCTB, as well as other primary bone tumors, if PSMA expression is found in its vasculature. This proof-of-concept study supports the justification and feasibility for the use of Pluvicto and Locametz in preclinical studies and randomized clinical trials focusing on the repurposing of commercially available PSMA-targeted diagnostic and therapeutic agents for the detection and treatment of GCTB and beyond.

## Data Availability

The raw data supporting the conclusions of this article will be made available by the authors, without undue reservation.
