# Supplementary material for: Repurposing of PSMA-targeted diagnostic and therapeutic agents for the detection and treatment of giant cell tumors of bone
Source: Front Oncol. 2024 Nov 15;14:1504514. doi: 10.3389/fonc.2024.1504514 (PMC11604636; doi:10.3389/fonc.2024.1504514)
Supplement: Supplementary file 1 [file DataSheet1.pdf]

Supplemental Figure 1

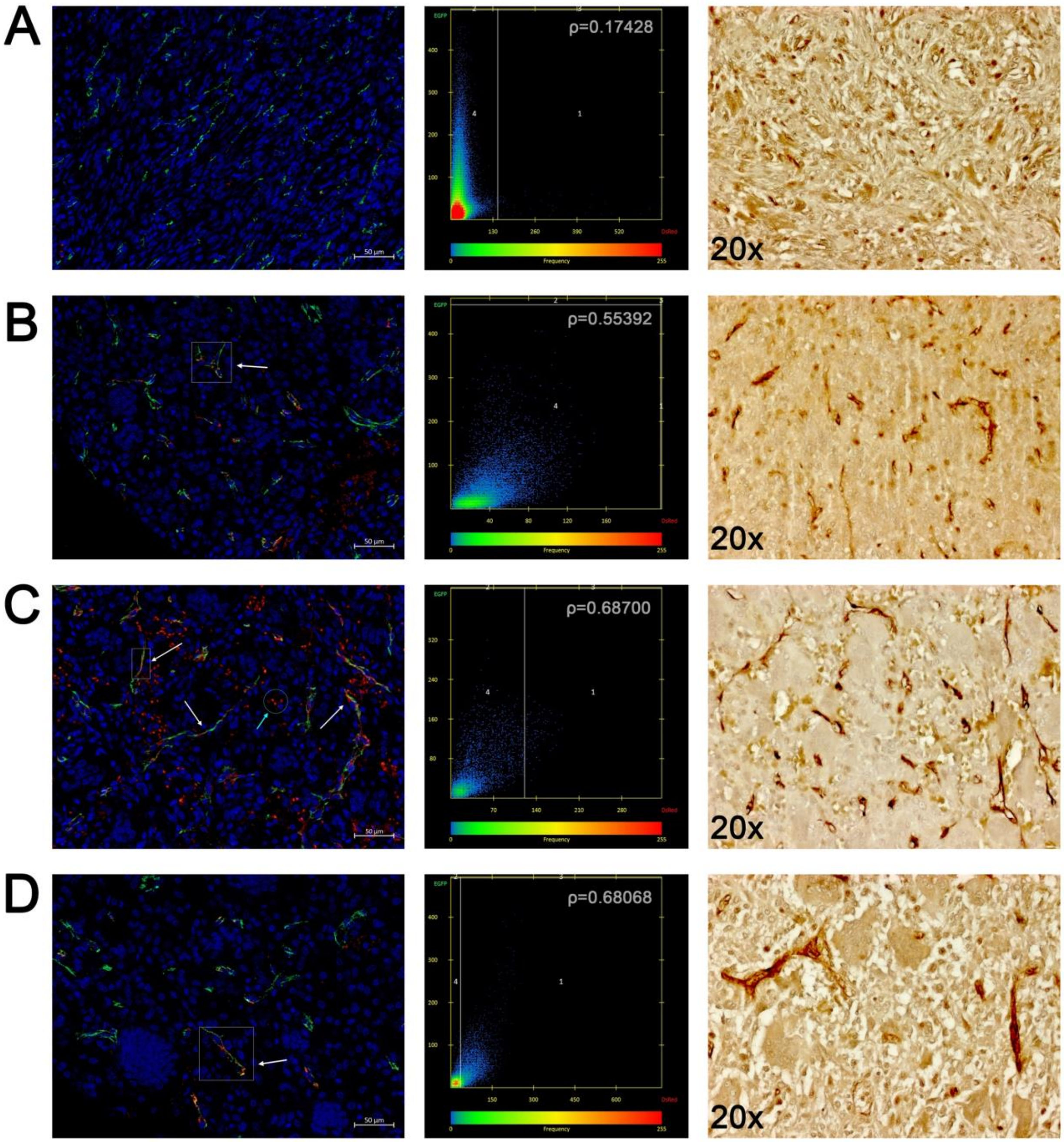

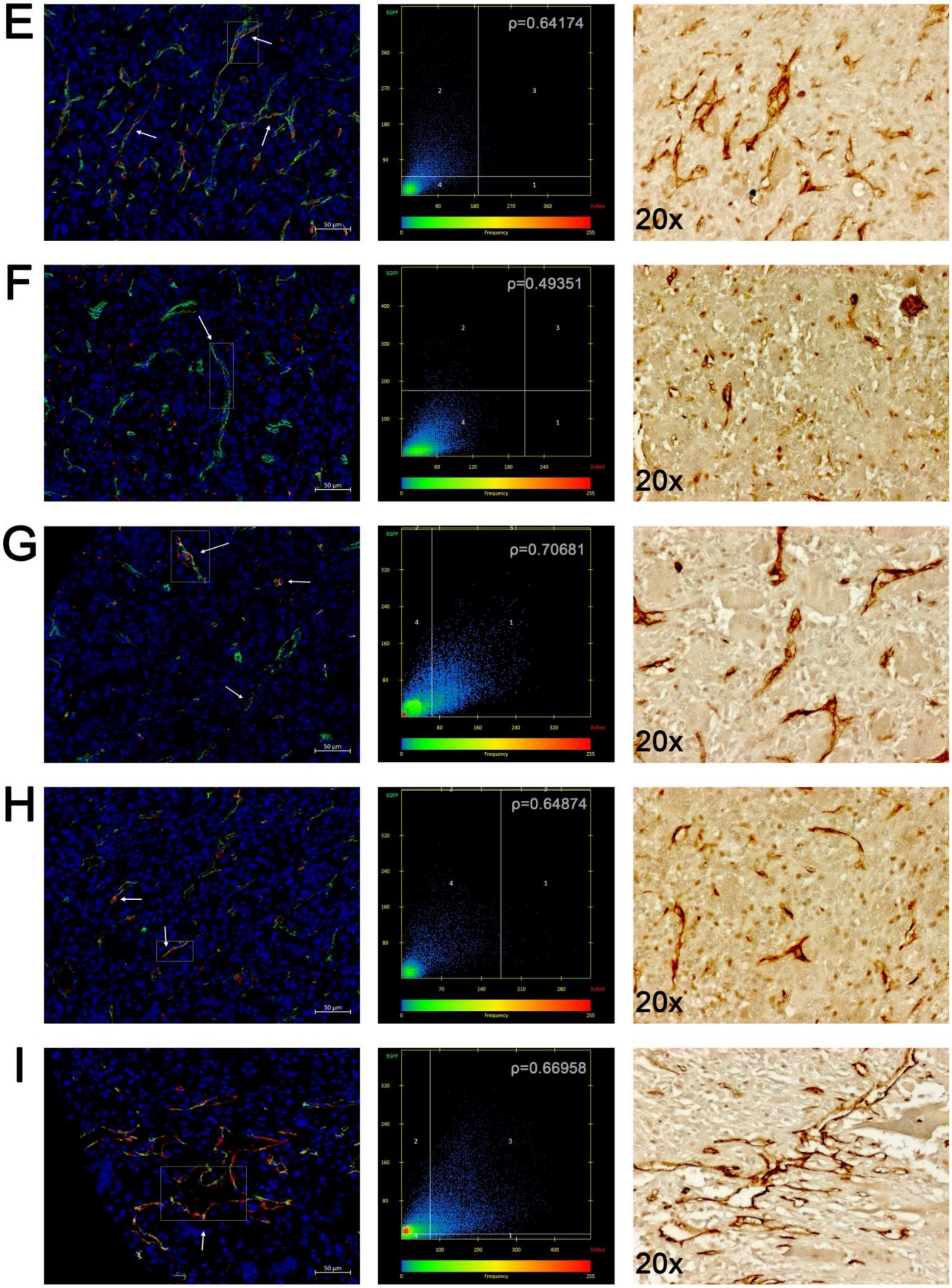

E
ρ=0.64174
20x
F
ρ=0.49351
20x
G
ρ=0.70681
20x
H
ρ=0.64874
20x
I
ρ=0.66958
20x

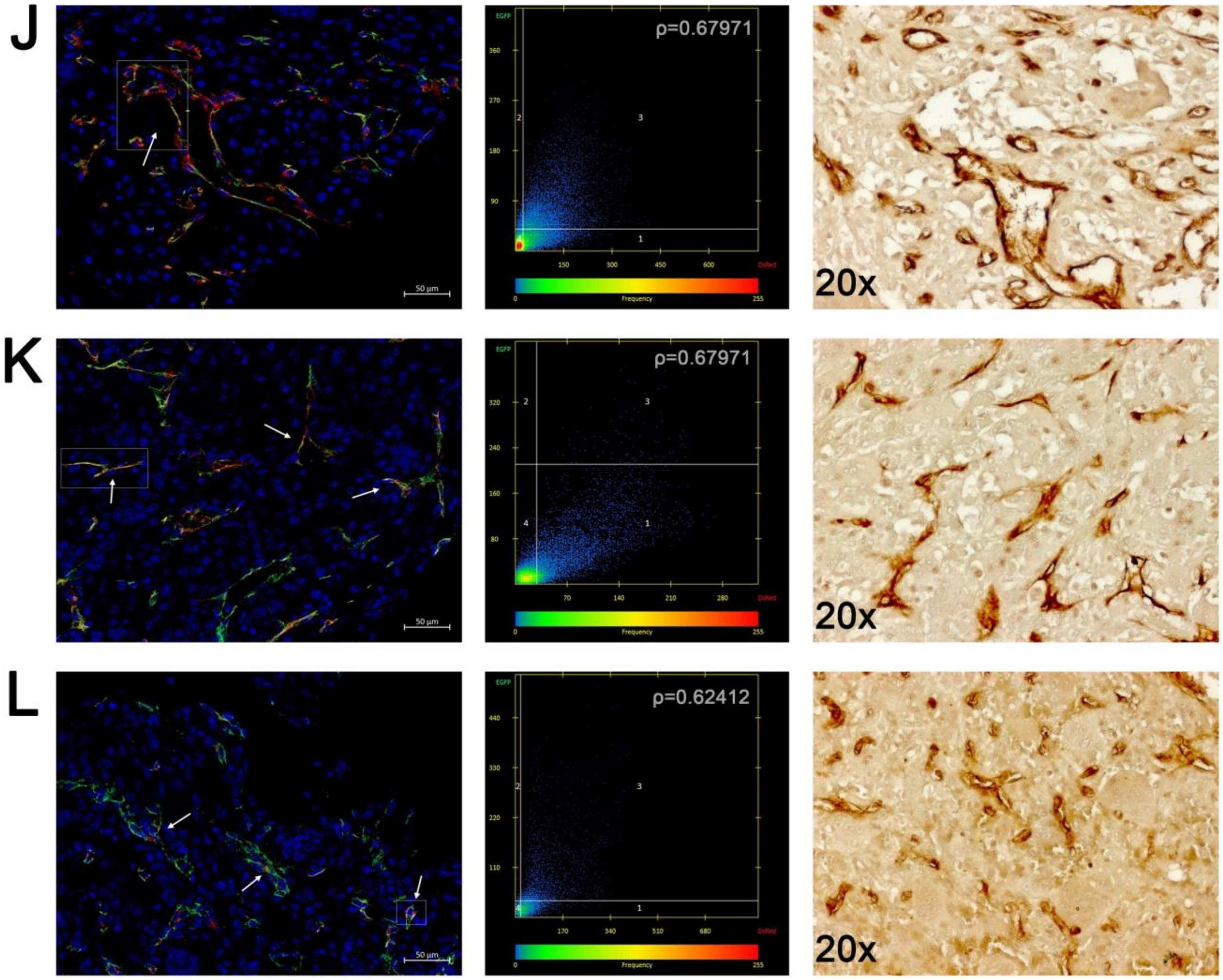
J
ρ=0.67971
20x
K
ρ=0.67971
20x
L
ρ=0.62412
20x
50 µm
EGFP
DsRed
Frequency

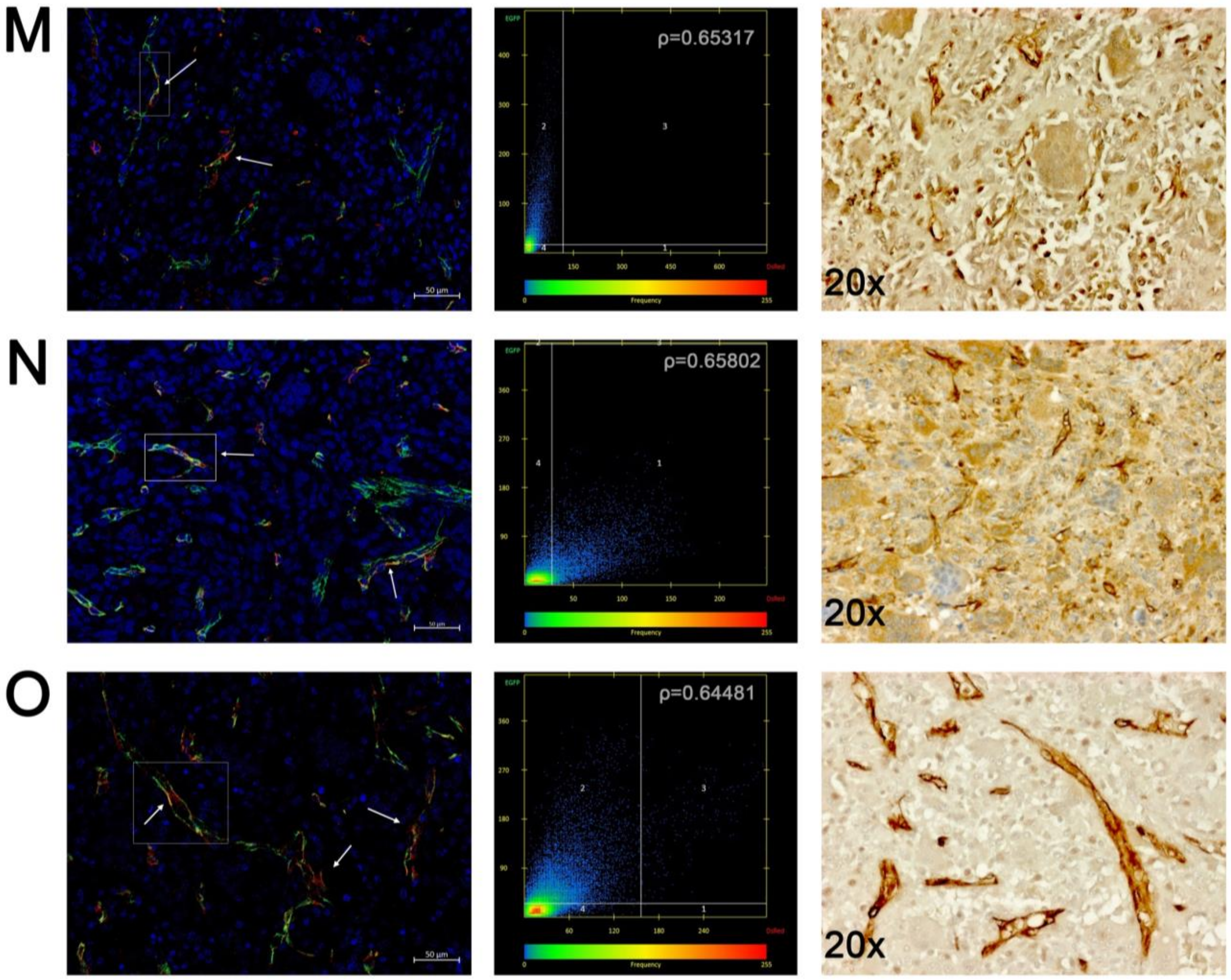

Supplemental Figure 1. PSMA is detected on the vasculature of GCTB male patients. **A-O**. IF staining PSMA (red), CD31 (green), and the nucleus (blue/DAPI) (20x, scale bar = 50μM) and matching colocalization scatter plot and IHC staining PSMA in brown (20x) for male patient ID#s 2-16. White arrows highlight areas of colocalization. Blue arrows and circles indicate the autofluorescence of red blood cells. The colocalization graph and Pearson correlation coefficient (ρ) as measured by Zeiss Zen software, correspond to the white-boxed areas where indicated. If no white box is present, the Pearson correlation coefficient (ρ) corresponds to the whole image. The intensity of a given pixel in the CD31 image is used as the y-coordinate of the scatter plot, and the intensity of the corresponding pixel in the PSMA is the x-coordinate.

Supplemental Figure 2

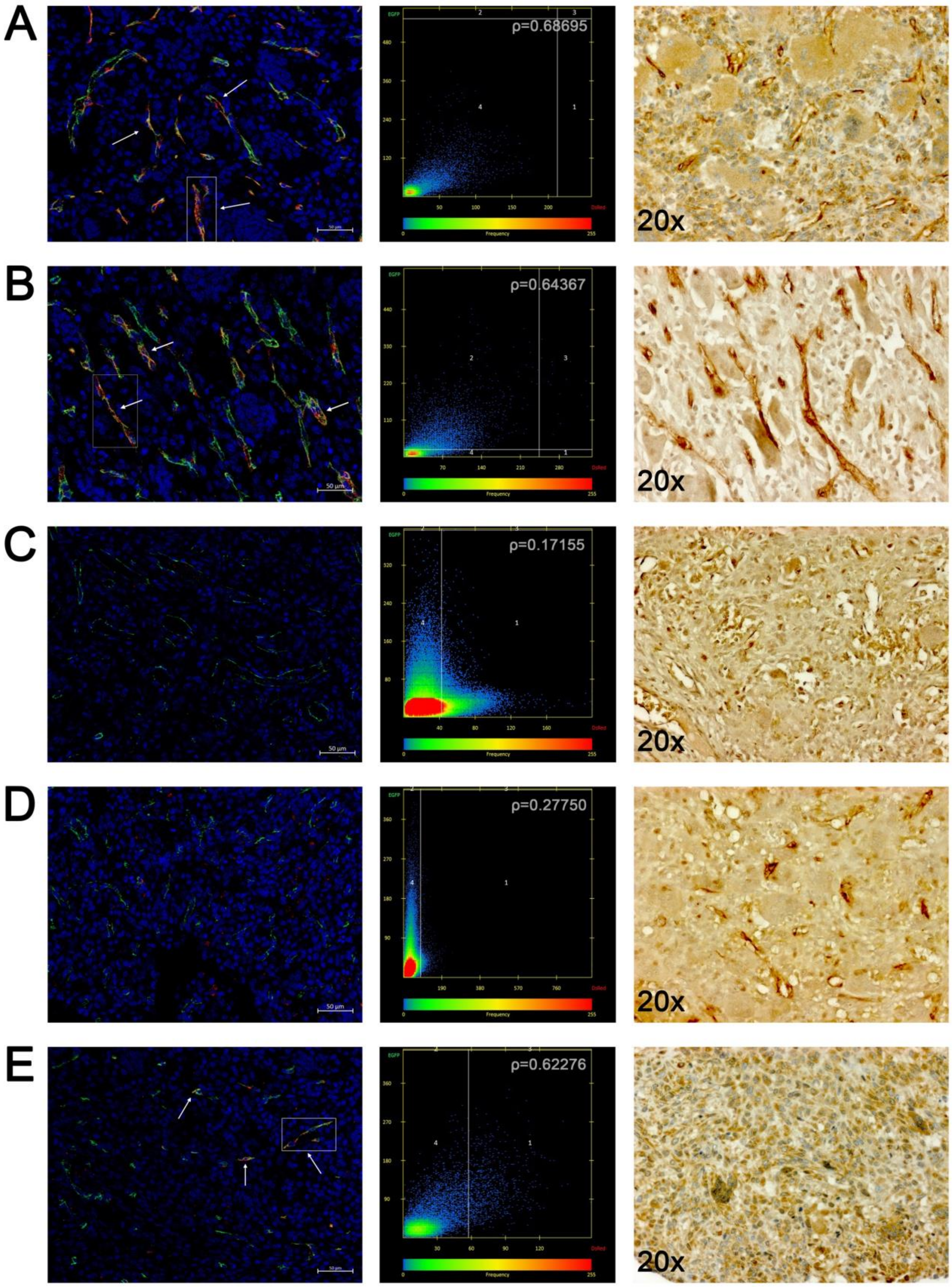

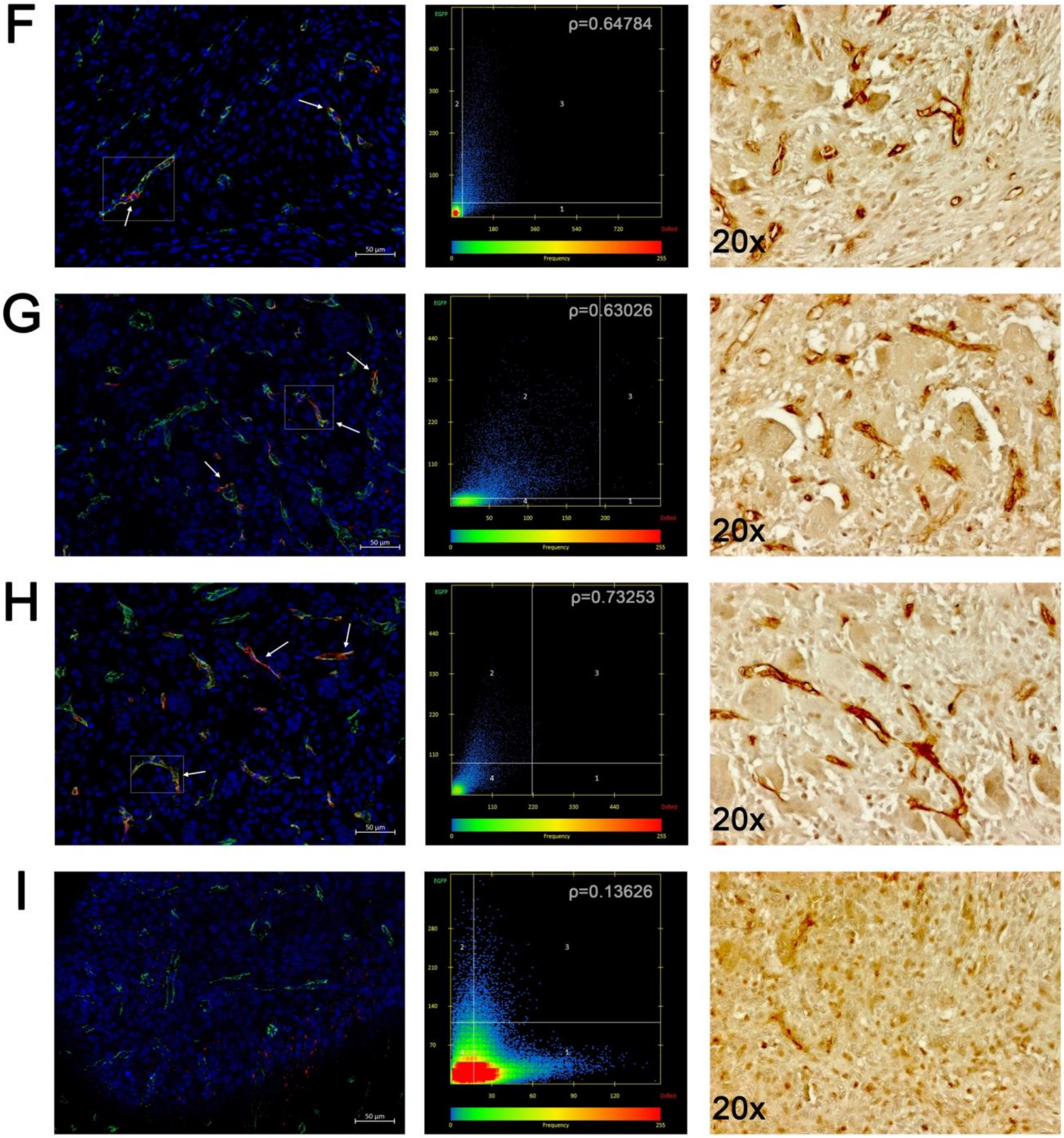

F
ρ=0.64784
20x
G
ρ=0.63026
20x
H
ρ=0.73253
20x
I
ρ=0.13626
20x

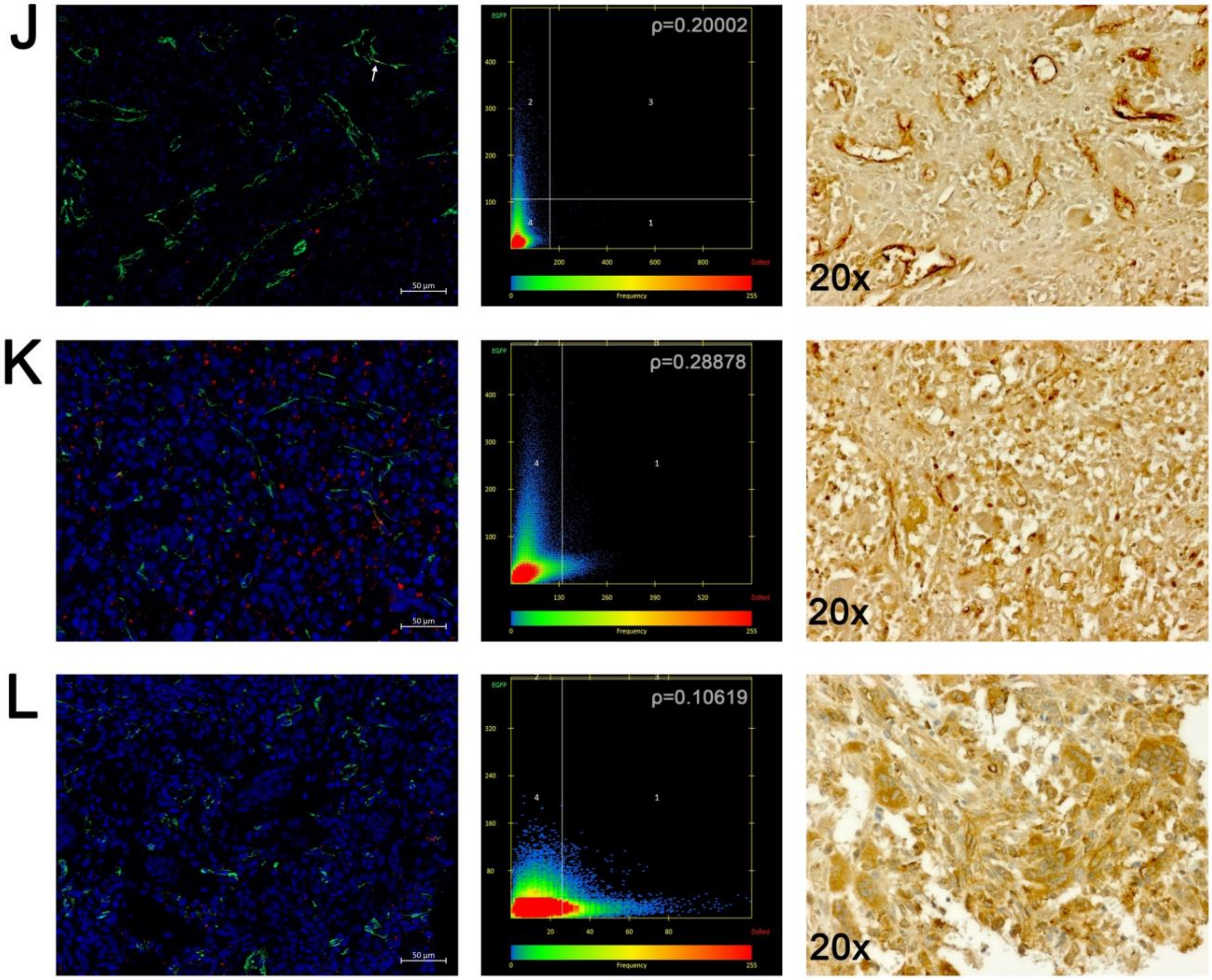

<u>Supplemental Figure 2.</u> PSMA is detected on the vasculature of GCTB female patients. **A-L**. IF staining PSMA (red), CD31 (green), and the nucleus (blue/DAPI) (20x, scale bar = 50μM) and matching colocalization scatter plot and IHC staining PSMA in brown (20x) for female patient ID#s 1-12. White arrows highlight areas of colocalization. The colocalization graph and Pearson correlation coefficient (ρ), as measured by Zeiss Zen software, correspond to the white-boxed areas where indicated. If no white box is present, the Pearson correlation coefficient (ρ) corresponds to the whole image. The intensity of a given pixel in the CD31 image is used as the y-coordinate of the scatter plot, and the intensity of the corresponding pixel in the PSMA is the x-coordinate.

Supplemental Figure 3

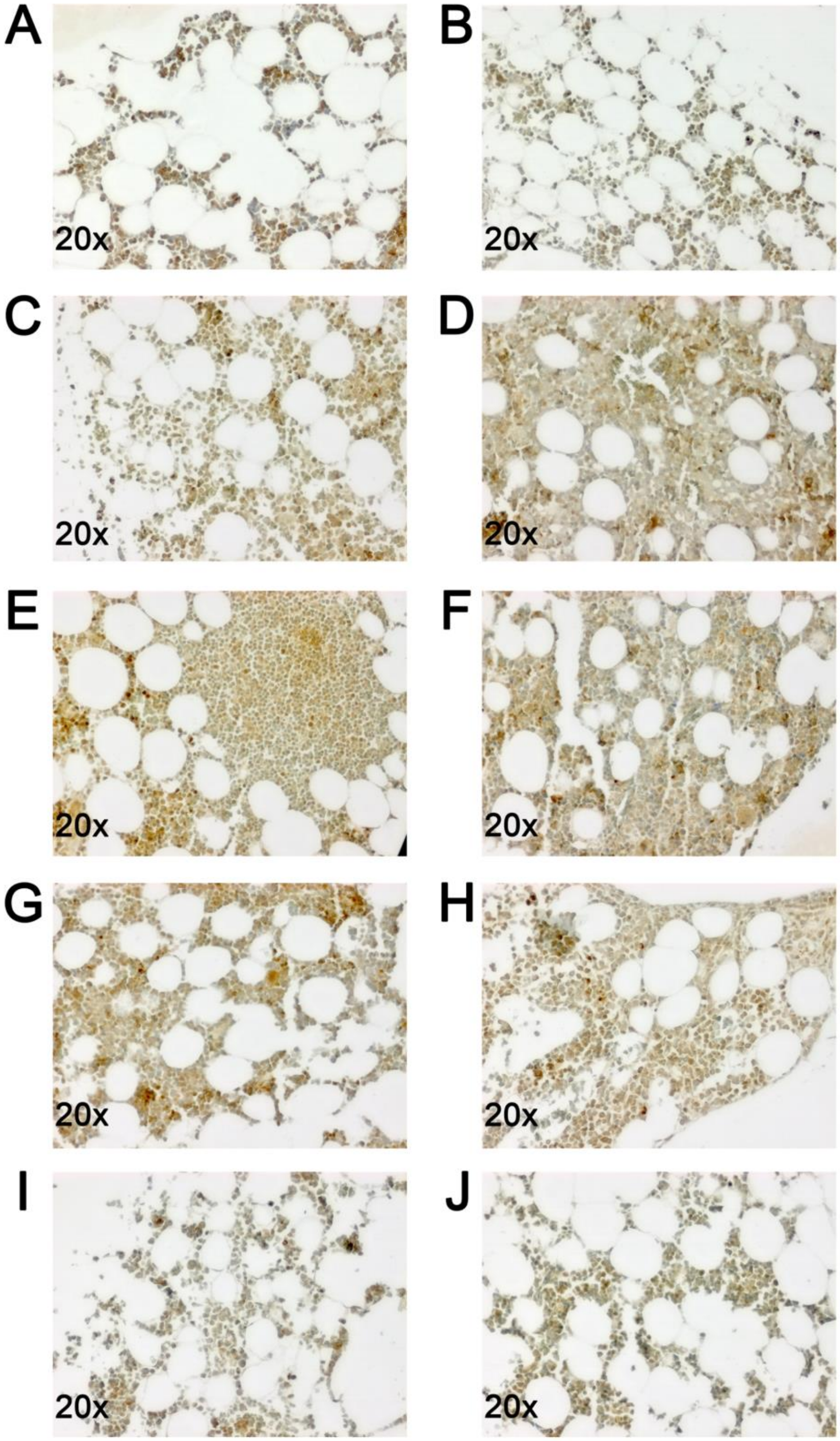

Supplemental Figure 3. PSMA is not detected in the vasculature of cancer adjacent to normal bone and bone marrow tissue. A-J. IHC staining of PSMA in cancer adjacent to normal bone and bone marrow tissue (NAT) of rib from negative control patient ID#s 1-10 (20x).

Supplemental Figure 4

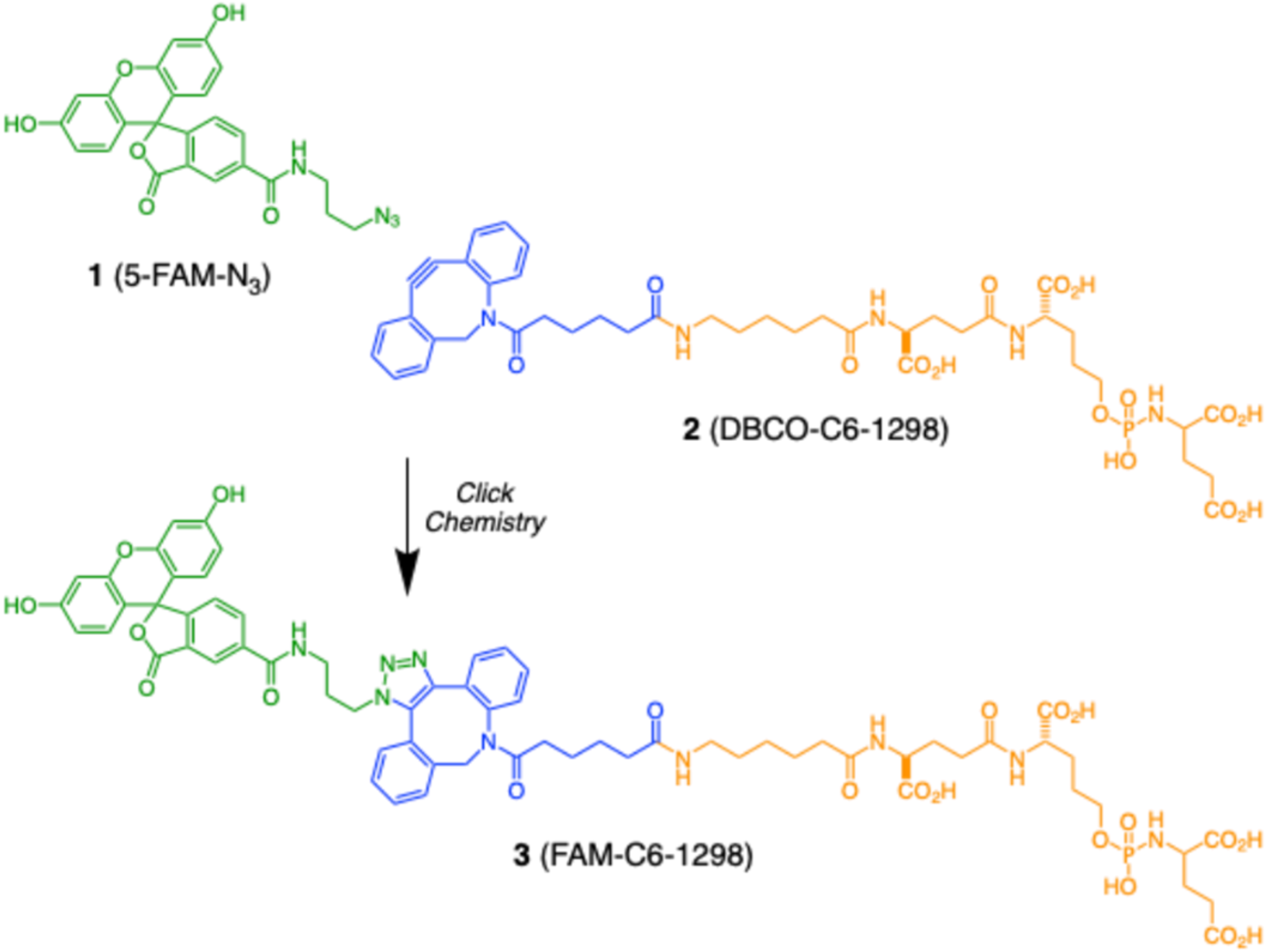

Supplemental Figure 4. Scheme 1 Modular assembly of PSMA-targeted fluorescent probe, FAM-C6-1298. A solution of compound **1**, 5-FAM-azide, was added to a solution of compound **2**, DBCO-C6-1298. Upon completion, excess compound **2** (DBCO-C6-1298) was removed using azide agarose resin to yield compound **3.**

Supplemental Figure 5. **Patient information from human GCTB samples purchased from OriGene.**

Catalog Number	Case ID	Age	Gender	Sample Type	Tissue of (Origin/Finding)	Appearance
CB649405	CU0000016174	47	Female	Frozen OCT-embedded Tissue	Bone: femur, distal / Bone: femur, distal	Tumor
CB649383	CU0000016173	23	Female	Frozen OCT-embedded Tissue	Bone: tibia / Bone: tibia	Tumor

Catalog Number	Case ID	Age	Gender	Sample Pathology from Pathology Verification	Tumor Grade	TNM
CB649405	CU0000016174	47	Female	Tumor of bone, giant cell	Not Reported	Not Reported
CB649383	CU0000016173	23	Female	Tumor of bone, giant cell	Not Reported	Not Reported

Catalog Number	Case ID	Age	Gender	Minimum Stage Grouping	% Normal	% Lesion
CB649405	CU0000016174	47	Female	Not Reported	0	0
CB649383	CU0000016173	23	Female	Not Reported	0	0

Catalog Number	Case ID	Age	Gender	% Tumor	% Tumor Hypercellular Stroma	% Tumor Hypo/Acellular Stroma
CB649405	CU0000016174	47	Female	98	0	2
CB649383	CU0000016173	23	Female	98	0	2

Catalog Number	Case ID	Age	Gender	% Necrosis	Sample ID	Case Diagnosis from Donor Institution Pathology Report
CB649405	CU0000016174	47	Female	0	FR0003F0D2	Tumor of bone, giant cell
CB649383	CU0000016173	23	Female	0	FR0003F092	Tumor of bone, giant cell

*OriGene provided patient information

Supplemental Figure 6.

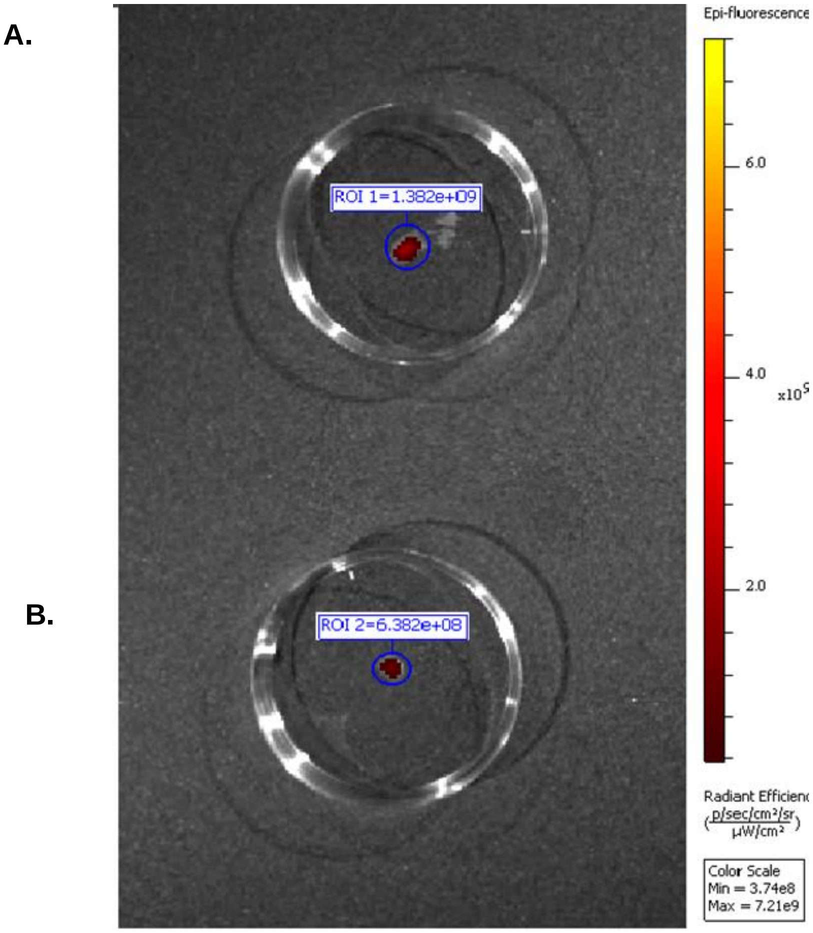

R0I	Total Radiant Efficiency (photon/s/cm^2/sr/µW/cm^2)	Average Radiant Efficiency (photon/s/cm^2/sr/µW/cm^2)	Background Corrected Intensity (photon/s/cm^2/sr/µW/cm^2)
1	1.38 x10^9	3.71 x10^8	1.01 x10^9
2	6.38 x10^8	2.95 x10^8	3.44 x10^8

Supplemental Figure 6. Figure 3: FAM-C6-1298 can successfully target PSMA in GCTB tissue. *Ex vivo* whole-tissue fluorescence measurements of fresh tissue from the tibia of a 47-year-old female with clinically diagnosed GCTB (Origene, catalog number CB649405) incubated with **A.** 10µM PSMA-targeted fluorescent probe, FAM-C6-1298. **B.** 100µM PSMA blocking peptide DBCO-C6-1298, followed by incubation with 10µM FAM-C6-1298. Data was collected by IVIS as radiant efficiency (photons/sec/cm^2/steradian/µW/cm^2) using Living Image software v4.8.2 and presented as a background-corrected intensity signal in the chart.

Supplemental Table 1

Female Patient ID	Yellow (colocalization) area	Green (CD31 individual) area	yellow:green% area
1	15788.66667	30774.66667	52.33
2	29729	66537	44.51
3	526	23800	2.14
4	559.5	21324	2.58
5	1684.666667	17721.33333	10.11
6	3319	18515.5	17.91
7	7224.5	36616	19.83
8	19741	38921.5	50.55
9	734	19345	3.95
10	731	31098	2.35
11	776.5	23663.5	3.22
12	811	15635.33333	4.97
**Male Patient ID**	**Yellow (colocalization) area**	**Green (CD31 individual) area**	**yellow:green% area**
1	5034	23523	15.41
2	331	24335.5	1.37
3	1792.5	17724	9.23
4	9380.333333	30393	31.19
5	2272	18008	12.65
6	11171	51602	18.16
7	1538	40521	3.82
8	3993	23490	16.99
9	4125	25633.5	16.35
10	7801	12847.5	58.14
11	16333	27732	58.90
12	13244	24482.5	56.99
13	2890	28682.5	9.54
14	3629	25473.33333	14.85
15	6825	24640.66667	25.24
16	7320.5	15392.5	38.49

Supplemental Table 1.
ImageJ colocalization analysis for GCTB patients. The percent area colocalization was determined using the color threshold function in ImageJ/Fiji. The area of PSMA colocalization was reported as a percentage of the area of CD31 staining, and the average percentage was reported from serial sections. The provided yellow and green area measurements are representative averages from serial tissue sections for simplification. >10% corresponds to PSMA positive (+) staining.

<u>Supplemental Table 2</u>

Sample (Treatment+Antibody)	Yellow (colocalization) area	Green(individual) area	Yellow:Green% area
FAM-C6-1298+EEA1 (endosome)	6771	64534	10.49
FAM-C6-1298+LAMP1 (lysosome)	30250	54928	55.07
FAM-C6-1298+PSMA	17003	32596	52.16
5FAM-X-FPO-42+PSMA	3584	5552	64.55

<u>Supplemental Table 2.</u>
ImageJ colocalization analysis for C42B-CRISPR-PSMAscramble cells treated with either FAM-C6-1298 or 5FAM-X-FPO-42. The percent area colocalization was determined using the color threshold function in ImageJ/Fiji. The area of colocalization with the endosome (EEA1), lysosome (LAMP1), or PSMA (yellow staining) was reported as a percentage of the area of FAM-C6-1298 or 5FAM-X-FPO-42 (green staining).

Supplemental Methods.

FAM-C6-1298 Synthesis: ^{1}H, ^{13}C, and ^{31}P NMR spectra were recorded on a Varian 400, Brucker Avance Neo 500, or Varian 600 MHz spectrometer. ^{1}H NMR chemical shifts are relative to $CDCl_3$ (δ = 7.26 ppm), CD_3OD (δ = 3.31 ppm) or D_2O (δ = 4.79 ppm). ^{13}C NMR chemical shifts were relative to $CDCl_3$ (δ = 77.23 ppm) or CD_3OD (δ = 49.15 ppm). ^{31}P chemical shifts were relative to triphenylphosphine oxide (TPPO, δ = 27.00 ppm). High-resolution mass spectrometry (HRMS) spectra were obtained on an Applied Biosystems 4800 MALDI-TOF/TOF mass spectrometer (Applied Biosystems, Foster City, CA).

A solution of **1**, 5-FAM-azide, (5 mg, 10.9 μmol) in MeOH was added to a solution of **2**, DBCO-C6-1298, (17.8 mg, 16 μmol) in 0.5 M $KHCO_3$ in ddH_2O. The reaction was stirred for 2 hours at room temperature and monitored by reverse-phase TLC. Upon completion, excess **2** (DBCO-C6-1298) was removed using azide agarose resin (Vector Laboratories, Newark, CA) (**Supplemental Figure 4).** The solvents were removed under reduced pressure, and the resulting residue was lyophilized overnight. The solids were desalted using C18 Sep-Pack (100:0 ddH_2O:ACN to 70:30 ddH_2O:ACN) to yield a yellow to orange solid at 41% yield. TLC: R_f = 0.60 (50:50 ddH_2O:ACN, visualization at 254 nm). ^{31}P NMR (162 MHz, D_2O) δ 4.70. HRMS (MALDI): m/z calculated for $C_{66}H_{72}N_9O_{21}P^-$ $[M+H]^-$: 1356.4502, found 1356.44885.

Reagents.

Patient Samples. Formalin-fixed paraffin-embedded (FFPE) bone core slides (TissueArray.com, Catalog #BO801, #BO601, and #T261b), GCTB tissue blocks (OriGene, catalog #CB499383, CB649405).

Cells. C4-2B (ATCC, catalog #CRL-3315), Gibco RPMI 1640 medium (Thermo Fisher, catalog #11875-093), fetal bovine serum (Corning, catalog #35-010-CV, Lot 17323001), Gibco antibiotic-antimycotic (Thermo Fisher, catalog #15240-062), and Gibco insulin-transferrin-selenium (Thermo Fisher, catalog # 41400045).

Antibodies – Primary and Secondary. PSMA (D4S1F) (Cell Signaling Technologies, catalog #2702), PSMA (D718E) (Cell Signaling Technologies, catalog #12815), CD31 89C2 (Cell Signaling Technology, catalog #3528), Alexa Fluor 546 goat anti-rabbit (Thermo Fisher Scientific, catalog #A11035), Alexa Fluor 488 goat anti-mouse (Thermo Fisher Scientific, catalog #A32723).

Immunofluorescence and Immunohistochemistry. 10 mM sodium citrate buffer (pH 6.0, EMD Millipore Corp, catalog #21545), Triton X-100 (Sigma Aldrich, catalog #93443), formalin solution, neutral buffered, 10% (Sigma-Aldrich, catalog #HT501128), Gibco phosphate-buffered saline (PBS) (Thermo Fisher, catalog #10010023), peroxidase suppressor (Thermo Fisher, catalog #35000), Gibco normal goat serum (Thermo Fisher, catalog #PCN5000), VECTASHIELD Hardset Antifade Mounting Medium with DAPI (Vector Laboratories, catalog # H-1500). Vector TrueVIEW (Vector Laboratories, catalog #SP-8400), VECTASTAIN Elite ABC Universal PLUS Kit, Peroxidase (Horse Anti-Mouse/Rabbit IgG Cat# PK-8200) (Vector Laboratories, catalog #PK-6101), Hematoxylin Gill's Formula (Vector Laboratories, catalog #H-3401-500), Cytoseal 60 (Epredia, catalog #8310-16).

Synthesis of FAM-C6-1298. DBCO-C6-1298 was available from a prior study (61) and 5-FAM-azide was purchased from Lumiprobe Corporation. All other reagents and general solvents were of commercial quality (Fisher Scientific, Sommerville, NJ) or (Sigma-Aldrich, St. Louis, MO) and were used without further purification. Anhydrous solvents used in reactions were obtained from commercial sources or freshly distilled over calcium hydride.
